# Unexpected Lack of Deleterious Effects of Uranium on Physiological Systems following a Chronic Oral Intake in Adult Rat

**DOI:** 10.1155/2014/181989

**Published:** 2014-02-12

**Authors:** Isabelle Dublineau, Maâmar Souidi, Yann Gueguen, Philippe Lestaevel, Jean-Marc Bertho, Line Manens, Olivia Delissen, Stéphane Grison, Anaïs Paulard, Audrey Monin, Yseult Kern, Caroline Rouas, Jeanne Loyen, Patrick Gourmelon, Jocelyne Aigueperse

**Affiliations:** ^1^Institut de Radioprotection et de Sûreté Nucléaire (IRSN), PRP-HOM, SRBE, LRTOX, 31 avenue de la Division Leclerc, BP 17, 92262 Fontenay-aux-Roses Cedex, France; ^2^Institut de Radioprotection et de Sûreté Nucléaire (IRSN), PRP-HOM, SRBE, LRTOX, BP 166, 26702 Pierrelatte Cedex, France; ^3^Institut de Radioprotection et de Sûreté Nucléaire (IRSN), PRP-ENV, STEME, BP 40035, 78116 Le Vésinet Cedex, France; ^4^Institut de Radioprotection et de Sûreté Nucléaire (IRSN), PRP-HOM, BP 17, 92262 Fontenay-aux-Roses Cedex, France

## Abstract

Uranium level in drinking water is usually in the range of microgram-per-liter, but this value may be as much as 100 to 1000 times higher in some areas, which may raise question about the health consequences for human populations living in these areas. Our purpose was to improve knowledge of chemical effects of uranium following chronic ingestion. Experiments were performed on rats contaminated for 9 months via drinking water containing depleted uranium (0.2, 2, 5, 10, 20, 40, or 120 mg/L). Blood biochemical and hematological indicators were measured and several different types of investigations (molecular, functional, and structural) were conducted in organs (intestine, liver, kidneys, hematopoietic cells, and brain). The specific sensitivity of the organs to uranium was deduced from nondeleterious biological effects, with the following thresholds (in mg/L): 0.2 for brain, >2 for liver, >10 for kidneys, and >20 for intestine, indicating a NOAEL (No-Observed-Adverse-Effect Level) threshold for uranium superior to 120 m g/L. Based on the chemical uranium toxicity, the tolerable daily intake calculation yields a guideline value for humans of 1350 *μ*g/L. This value was higher than the WHO value of 30 *μ*g/L, indicating that this WHO guideline for uranium content in drinking water is very protective and might be reconsidered.

## 1. Introduction

Uranium is a natural component of the earth crust. Its concentrations vary highly according to the location and the type of rocks [1]. In fact, it can vary from 3 to 4 g/t in granites and from 20 to 120 g/t in phosphate rocks [2], leading consequently to a great variability of the uranium content in drinking water. For instance, average uranium levels in drinking water are in microgram-per-liter range (0.4 *μ*g/L throughout the world, [3]; 6.7 *μ*g/L in USA, [4]; 4 *μ*g/L in Canada [5]; 2.2 *μ*g/L in China [6]; or 2.22 *μ*g/L in France [7], but they may reach exceptionally the milligram-per-liter range in some specific areas, including some US states (2.5 mg/L [4]; 7.8 mg/L [8]) or southern Finland (9.2 mg/L [9]; 3.4 mg/L [10]). Anthropogenic utilization of uranium for civil, military, and nuclear fuel purposes leads to additional release of this radio element into environment, either around uranium mining sites [11, 12], uranium reprocessing plants [13], or following the use of depleted uranium (DU) in conflict zones [11, 14]. The persistent use of depleted uranium in weapon composition in the recent conflicts (Iraq in 1991 and 2001, Bosnia and Herzegovina in 1995, and Kosovo in 1999) continues to keep the scientific and social queries concerning the environmental contamination by such metal, as well as subsequent human exposure and possible consequences on health.

The particularity of uranium is its dual toxicity, due to its radiological effects as an alpha emitter and its chemical effects due to its properties as metal. However, the chemical toxicity of this radionuclide was considered as predominant in case of depleted and natural uranium. The major biological effects of uranium on health concern renal function [15, 16]. According to these specific properties, most of guidelines concerning uranium levels were based on chemical effects of uranium on kidneys. As ingestion (by drinking water or alimentary chain) is the predominant way of human exposure for public in case of environmental exposure, guideline values concerning uranium level in drinking water were established by several national and international organizations. These guidelines were based on existing information about chemical effects of uranium, mainly in kidneys. Thus, the World Health Organization set a guideline value of 15 *μ*g/L for uranium in drinking water [3], recently reevaluated at 30 *μ*g/L [17]. This value, however, was provisional in view of several limitations pointed out by WHO [17] concerning the lack of extensive knowledge about the chronic effects of uranium in drinking water. These restrictions were in accordance with a recent review that claimed the need for more epidemiological and experimental studies [18]. Indeed, the existing epidemiological studies do not allow clearly demonstrating health effects of radionuclides at levels naturally encountered in drinking water due to methodological limitations (exposure assessment, possible confounders, and limited sample size). In addition, few experimental studies were performed with uranium levels close to real environmental situations. Considering the previous guideline of WHO [3], the value of 15 *μ*g/L was derived from a single study of subchronic renal effects of uranium when administered to rats in drinking water for 3 months [19]. Moreover, several authors have since shown that the kidneys are not the only biological target of chronic exposure to low levels of uranium; for examples changes have been reported in the gastrointestinal system [20], in the central nervous system [21, 22], or in the liver [23].

These two points—the limitations of existing studies and necessity to take into consideration the recent concept that uranium affects multiple organs—led us to conduct new experimental studies of the results of chronic uranium exposure. The investigation of this present study focused on five organs/tissues: the small intestine, which is the entry route for uranium after ingestion [24]; the kidneys, traditionally considered the target organ of uranium following an acute contamination at high doses [16]; the hematopoietic cells that may be modified by uranium due to its accumulation in the bones and kidneys [25], and other organs described more recently as affected by chronic exposure at low levels: the liver [23] and the brain [22]. The objective was to evaluate the possible biological effects of uranium on the primary function of each organ/tissue. Concerning the intestines, we choose to investigate uranium effects on immune function of intestinal mucosa, because previous studies demonstrated a specific localization of uranium in immune cells of intestinal wall [26, 27]. The investigation of uranium effects on the kidneys and the liver was based on the different phases of the xenobiotic metabolism, since they are the major organs involved in detoxification processes of endo- and xenomolecules. In addition, cholesterol metabolism was studied in the liver, since this organ plays the central role in whole organism concerning this metabolism. The effects of uranium on the central nervous system were evaluated *via* two mechanisms, the uranium-induced oxidative stress and the cholinergic system. Indeed, a possible mechanism of uranium effects observed in cognitive functions may be due to the oxidative stress induced by reactive oxygen species imbalance.

In order to complete this investigation and to be sure to provide an extensive view of uranium effects in the present study, the analyses were performed at the several macroscopic and microscopic levels of organism (molecular, functional, structural, and pathological).

This study was performed in rats of a wide range of concentrations (0.2, 2, 5, 10, 20, 40, or 120 mg/L) of uranium in drinking water. These uranium levels include the uranium level of 0.2 mg/L close to values measured around mining areas [28], the uranium level of 1 mg/L close to the concentration of 0.96 mg/L that served to determine the previous WHO guideline of uranium level in drinking water [3], and the maximal concentration measured throughout the world (20 mg/L in Finland [29]). Duration of 9 months was chosen for the present study to mimic the chronic exposure of populations living on uranium-rich territories and to evaluate its long-term consequences.

## 2. Material and Methods

### 2.1. Animals

These experiments were conducted on 8-week old Sprague-Dawley male rats (Charles River, L'Arbresle, France) according to French regulations for animal experimentation (Ministry of Agriculture Act number 2001-464, 2001) and with the approval of IRSN animal welfare committee. Two rats were housed per cage at 21 ± 2°C and a 12 h : 12 h cycle with free access to normal rat food (105, CERJ, France) and water. Animals were contaminated for 9 months with drinking water containing uranium (uranyl nitrate hexahydrate (UO_2_(NO_3_)_2_·6H_2_O)) dissolved in mineral water for final concentration at 0.2, 2, 5, 10, 20, 40, or 120 mg/L (AREVA, France) (10 rats per group). The group of 40 mg/L was added in this study to serve as the reference group, as several studies have been published with this uranium level in drinking water [20, 23, 30, 31]. Control animals drunk uncontaminated mineral water (which contains naturally uranium at 1.42 *μ*g/L). Alpha spectrometry following chemical separation was performed to determine uranium isotope ratio in the contaminated water according the standard norm NF M 60-805-5. The ^235^U/^238^U ratio of 0.25% indicated depleted uranium (Table 1), which was used in this study to address the chemical toxicity of this radionuclide. At the end of contamination, after overnight fasting, the animals were anesthetized by inhalation of 5% isoflurane (Abbot France, Rungis, France) before being euthanized for tissue sampling. These experiments were conducted on male animals, since a previous study demonstrated that this gender in rats is more sensitive to uranium than the female is [19].

### 2.2. Uranium Measurements in Biological Samples

Biological samples were prepared by adding 8 mL nitric acid 70% ultra-pure (INSTRA-analyzed for trace metal analysis, Sodipro, Echirolles, France) and 2 mL of hydrogen peroxide. They were then mineralized using a 1000 W microwave (Ethos Touch, Milestone Microwave Laboratory Systems, Sorisole, Italy). Before measurement, samples were diluted between 1/5 and 1/500 depending on considered organ and administrated dose in 2% nitric acid. The samples were then analyzed for their uranium content by inductively coupled plasma mass spectrometry (ICP-MS) (XSERIE 2, Thermoelectron, France). Calibration was performed with a multielemental standard solution (Analab-STD-495, Analab, Bischheim, France). In all solutions likely to be analyzed (biological samples or calibration solutions), bismuth 209 was added as an internal standard. Further indications have been provided in a previous study [32]. For ^238^U, the detection and quantification limits were, respectively, 0.5 ng/L and 1.5 ng/L, and for ^235^U, 0.01 ng/L and 0.03. The limits for ^238^U were applied to total uranium. Uranium was measured in kidney, bone (femur), liver, and brain (entorhinal cortex).

### 2.3. Biochemical Parameters in Blood

An automated spectrophotometric system (Konelab 20, Thermo Electron Corporation, Cergy-Pontoise, France) was used to measure these parameters, with biological chemistry reagents from the manufacturer or Diagam (Lille, France). Bilirubin was measured with the Thermo Electron Corporation kit. Kits from Diagam were used for measuring total cholesterol, low-density lipoprotein- (LDL-) cholesterol, high-density lipoprotein- (HDL-) cholesterol, triglycerides, alanine aminotransferase (ALT), aspartate aminotransferase (AST), creatinine, urea, iron, ferritin, transferrin, and ceruloplasmin. Phospholipids B were measured with a kit provided by Diagnostic Partners (Bougival, France).

### 2.4. Blood Cell Counts

Blood was harvested by intracardiac puncture into either EDTA coated tubes for plasma isolation or dry tubes for serum isolation. Blood cell counts were performed by means of an MS-9 vet automatic counter (Melet-Schlossing, Osny, France). The remaining blood was centrifuged at 400 g for 10 minutes and plasma or serum was frozen for later use.

### 2.5. Cytokine Measurements

Flt-3 ligand concentration, a biological indicator of bone marrow function [33], and IL-7, a biological indicator of T lymphocyte homeostasis [34], were measured in plasma samples using ELISA kits according to manufacturer's recommendation (R&D System, Abington, UK). Sensitivity was 7 pg/mL for Flt3-ligand ELISA test and 15 pg/mL for IL-7 ELISA test.

### 2.6. Colony-Forming Cell Assay

Femurs were flushed with 5 mL washing medium (MEM-*α* medium supplemented with 1% fetal calf serum (FCS) and antibiotics, both from Life Technologies, Cergy-Pontoise, France). Spleen cells were crushed in Tenbrock's Potter in the presence of 5 mL washing medium. After washing twice (8 min at 400 g), cells were numbered and viability assessed in the presence of Trypan Blue. Cells were then plated at 5 × 10^5^ for spleen cells and 5 × 10^4^ bone marrow cells in 1.1 mL of complete methylcellulose medium with cytokines (Stem Cell Technologies, Vancouver, Canada). Cultures were incubated at 37°C in 95% air/5% CO_2_ in a humidified atmosphere. Colony-forming units-granulocyte macrophage (CFU-GM) and burst-forming units-erythroid (BFU-E) were scored on day 12 of culture.

### 2.7. Histology and Immunohistochemistry

After fixation in 4% formaldehyde solution (Carlo Erba, Rueil Malmaison, France), tissues were dehydrated, embedded in paraffin, and cut in sections 5 *μ*m thick. Hematoxylin-eosin-saffron staining was then performed in sections of liver, kidney, and terminal ileum by an independent laboratory (Biodoxis, Romainville, France).

Determination of immune cells was also performed in terminal ileum by immunohistochemistry or histochemistry as previously reported in [20]. The tissue neutrophil infiltration was estimated on paraffin sections incubated with rabbit antibody directed against rat myeloperoxidase (IMG antibody, *d* = 1/*x*00, Clinisciences, Nanterre, France) and LSAB2 HRP kit (Dako, Trappes, France). The macrophages were visualized with mouse antibody against CD68 (Serotec, Cergy Saint Christophe, France) followed by histofine simple stain mouse MAX PO (Microm Microtech, Francheville, France). For mast cell coloration, the ileal segments were fixed in Carney solution. Histological slides were then stained with Alcan Blue method. Neutrophils and mast cells were quantified by determining the number of their respective cells per crypt-villus axis. The results were expressed as mean ± SD of 8 animals. A qualitative analysis was realized for macrophages with establishment of a scoring of macrophage density network in crypts and villi.

### 2.8. Gene Expression

The different genes measured in the different organs were indicated in the Table 2. Total RNA was prepared from tissues (~30 mg for liver, kidney, entorhinal cortex, and ileum) with the RNeasy Mini Kit (Qiagen, Courtaboeuf, France) following the manufacturer's instructions. The NanoDrop apparatus (ThermoFisher Scientific, Cergy Pontoise, France) was used for determining the concentration of RNA in ng/*μ*L. For the reverse transcription, 1 *μ*g of total RNA was reverse transcribed at 37°C for 120 minutes using the High Capacity cDNA Reverse Transcription kit (Applied Biosystems, Courtaboeuf, France) according to the manufacturer's instructions. Real-time semiquantitative analysis was performed with the Abi PRISM 7900 sequence detection system (Applied Biosystems). The PCR amplification was performed using Syber PCR master mix (Qiagen) in a final volume of 25 *μ*L. Sequences for the forward and reverse primers of different genes studied in the present study are listed in Table 2. The relative mRNA quantification of the target gene was made by using the comparative ΔΔCT-method [35]. Data were first normalized to an endogenous reference (HPRT: hypoxanthine-guanine phophoribosyltransferase, a housekeeping gene) and expressed as the level relative to the uncontaminated controls (mean ± SD; *n* = 10).

### 2.9. Determination of Inflammatory Mediators in Intestine

Mucosal samples were obtained by scraping the rat terminal ileum at euthanasia and were kept at 80°C until analysis. Mucosal protein extracts were obtained from tissue homogenates performed using ribolyser (FastPrep 120, ThermoSavant, ThermoScientific, Cergy Pontoise, France) in phosphate buffer (pH = 7.4, PBS, Gibco, Invitrogen, Cergy Pontoise, France) containing a protease inhibitor cocktail (0.5 mL/100 mL, Sigma, L'Isle D'Abeau Chesnes, France). This step was followed by a centrifugation step (10000 g, 10 minutes). Tissue levels of cytokines were measured by ELISA assays: TNF*α*, IFN*γ*, and IL-10 were measured using Duoset kit ELISA (R&D systems, Lille, France) and CCL-2 using the Kit rat MCP-1 (Clinisciences, Montrouge, France). To ensure a higher sensitivity and a much greater dynamic range than that of classical colorimetric revelation by tetramethylbenzidine (TMB) substrate, a chemiluminescent substrate (luminol/peroxide substrate) is added to the wells (Glo Substrate, R&D systems, Lille, France). The results were expressed per mg of protein determined with Bradford assay (Sigma, L'Isle D'Abeau Chesnes, France).

### 2.10. Expression of Enzymes of Xenobiotics Metabolism Measured by Western Blotting on Liver and Kidney Samples

Proteins from tissue homogenates (microsome or cytosol) were prepared as described in [23]. These proteins were loaded, separated by 10% SDS-polyacrylamide gel electrophoresis and transferred onto nitrocellulose membrane. The membranes were blocked for 1 h in 5% nonfat dry milk in TBS. The blots were incubated overnight with antibodies diluted in 2% nonfat dry milk in TBS at 4°C. Microsomal CYP3A1 and CYP3A2 were detected using rabbit polyclonal antibodies (Abcam, Paris, France). Microsomal CYP2C11 was detected using goat polyclonal antibodies (Daiichi Pure Chemicals, Tokyo, Japan). Measurements of microsomal (CYP3A1, CYP3A2, CYP2C11, and UGT2B1) and cytosolic (GST2a) enzymes were detected and measured as described [36]. Microsomal UGT2B1 was detected using goat polyclonal antibodies and rabbit polyclonal antibodies, respectively (Santa Cruz Biotechnology, Heidelberg, Germany). Cytosolic GST2a was detected using goat antibody (Oxford Biomedical Research, Oxford, USA). Immune complexes were revealed by rabbit anti-goat IgG and goat anti-rabbit IgG (Santa Cruz Biotechnology) coupled to horseradish peroxidase (HRP) and the luminol derivative of Immobilon Western (Millipore, Billerica, USA). Samples were normalized to glyceraldehyde 3-phosphate dehydrogenase (GAPDH) which was detected using rabbit anti-rat antibody (Santa Cruz Biotechnology). Reaction intensity was determined by computer-assisted densitometry (Fuji Las3000, Raytest, France). Samples were normalized to glyceraldehyde 3-phosphate dehydrogenase (GAPDH).

### 2.11. Xenobiotic Metabolism Enzyme Activity in the Liver

Freshly prepared microsomal fractions were used to measure testosterone hydroxylase activity [36]. The enzymatic activity of 2*α*-testosterone hydroxylase (CYP2C11) and 6*β*-testosterone hydroxylase (CYP3A1/2) was expressed as picomoles per minute per whole liver and normalized to that of the noncontaminated group.

### 2.12. Cholesterol Metabolism Enzyme Activity in the Liver

The specific activities of cytochromes P450 CYP27A1 and CYP7A1 were assessed, respectively, on hepatic mitochondrial and microsomal fractions, with a radioisotopic method [37]. Enzymatic activity was expressed as picomoles per minute per milligram. The results are expressed as mean ± SEM of 6 animals.

### 2.13. Antioxidant Status in the Brain

The tissues were prepared as described in [30]. Each cerebral sample was measured in duplicate. The tissues were homogenized in ice-cold 0.1 M phosphate-buffered saline (PBS, pH 7.4) containing 1 mM EDTA. The homogenates were centrifuged at 10000 g for 15 min at 4°C, and the supernatants were collected for analyses. Protein concentrations were determined by Bradford method using bovine serum albumin as a standard. The activity of CAT (catalase), SOD (superoxide dismutase), glutathione peroxidase (GPx), and total glutathione (GSH) was determined with commercial kits (Bertin Pharma, Montigny-le-Bretonneux, France).

For CAT activity, the method is based on the reaction of the enzyme with methanol in the presence of an optimal concentration of H_2_O_2_. The formaldehyde produced is measured spectrophotometrically with 4-amino-3-hydrazino-5-mercapto-1,2,4-triazole (Purpald) as the chromogen. The CAT activity was expressed in nmol of formaldehyde per min per mg protein.

SOD activity is assessed by using a tetrazolium salt for detection of superoxide radicals generated by xanthine oxidase and hypoxanthine. The SOD assay measures all three types of SOD (Cu/Zn-, Mn-, and Fe-SOD). One unit of SOD is defined as the amount of enzyme needed to exhibit 50% dismutation of the superoxide radical. The enzyme units were recorded as U per mg protein.

GPx activity was indirectly measured by a coupled reaction with glutathione reductase (GR). Oxidized glutathione (GSSH), produced upon reduction of hydroperoxyde by GPx, is recycled to its reduced state by GR and NADPH. The oxidation of NADPH to NADP^+^ is accompanied by a decrease in absorbance at 340 nm, which is directly proportional to the GPx activity. The enzyme units were recorded as nmol NADPH oxidized per min per mg protein.

Cayman's GSH assay kit utilizes a carefully optimized enzymatic recycling method, using glutathione reductase for the quantification of GSH. The rate of TNB (5-thio-2-nitrobenzoic acid), produced by the reaction of the sulfhydryl group of GSH with DTNB, is directly proportional to the recycling reaction which in turn is directly proportional to the concentration of GSH. Measurement of the absorbance of TNB at 405 nm provides thus an accurate estimation of GSH. GSH is easily oxidized to the disulfide dimer GSSG. Both GSH and GSSG are measured in this assay kit and the assay reflects thus total glutathione.

### 2.14. Cholinergic Pathway in the Cerebral Cortex

Acetylcholine rates and acetylcholinesterase activity were measured in the entorhinal cortex with the Amplex Red Acetylcholine/Acetylcholinesterase Assay Kit (Molecular Probes, Invitrogen, Cergy Pontoise, France) as described in [38]. For both assays, samples of ~30 mg were diluted in Tris-HCl buffer (final volume of 100 *μ*L). A 100 *μ*L volume of 400 *μ*M Amplex Red Reagent containing 2 U/mL of HRP, 0.2 U/mL of choline oxidase, and 1 U/mL acetylcholinesterase was added to samples. Results were expressed as *μ*M/*μ*g protein. Absorbance was measured at 595 nm following a 45 min incubation at room temperature. The acetylcholinesterase assay was performed with a working solution of 200 *μ*M Amplex Red Reagent containing 2 U/mL of HRP, 0.2 U/mL of choline oxidase, and 100 *μ*M of acetylcholine. Absorbance was measured at 595 nm after 30 minutes of incubation at room temperature. Results were expressed as *μ*mole/mg protein/hour.

### 2.15. Statistical Analysis

Results are presented as mean ± SD from 5 to 10 animals, depending on the parameters measured. The control and each of the contaminated groups were compared with one-way ANOVA analysis and the post hoc Holm-Sidak method. Statistical significance was defined by a *P* value ≤0.05. All statistical analyses were performed with SigmaStat, Statistical Software (SPSS, Paris, France).

## 3. Results

### 3.1. Health Parameters: Body Weight Gain, Food Intake, and Water Consumption

**Figure 1:**
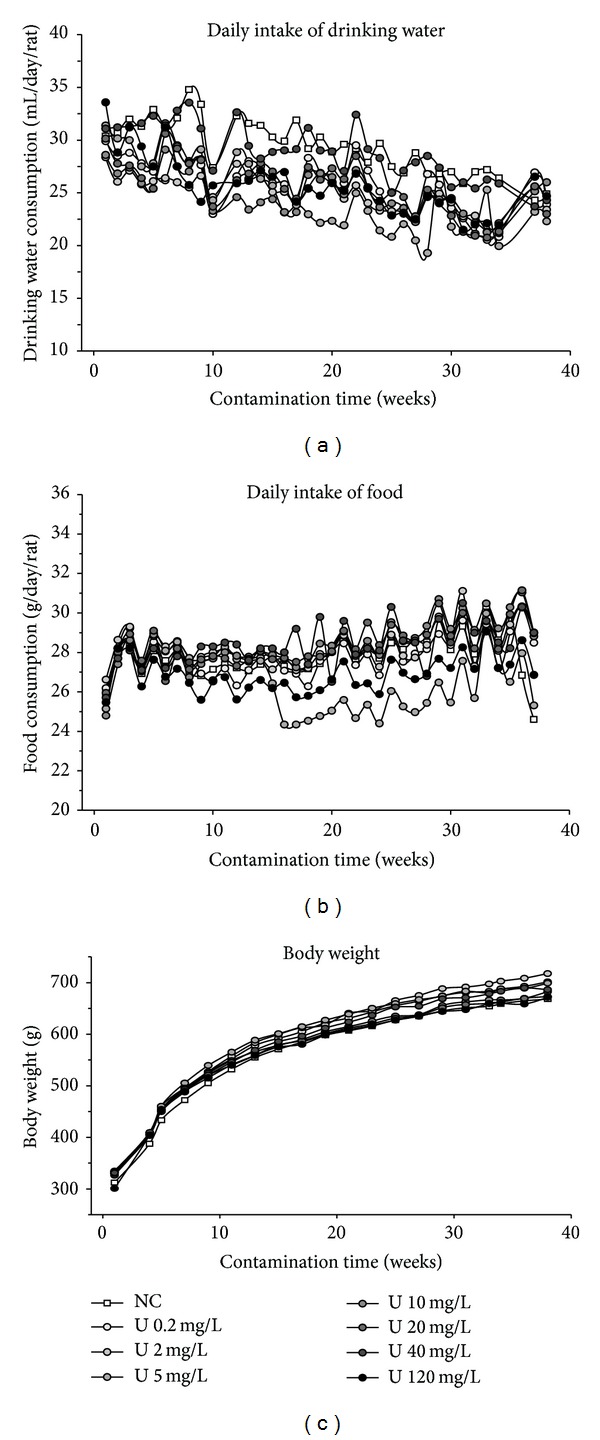
Effect of chronic ingestion of uranium on body weight, food, and drinking water intakes. Three parameters were recorded weekly in control group (open square) and in animals contaminated with uranium at 0.2, 2, 5, 10, 20, 40, or 120 mg/L (close circles). For standardization, drinking water consumption was expressed as mL per day per rat, food consumption was expressed as g per day per rat, and body weight was expressed by g. The indicated values are mean of *n* = 10 animals for body weight and *n* = 5 for daily intake of food and drinking water (two rats were housed per cage). For a clearer visualization, the SD of these values were not indicated in this figure: they were below 30% of mean values. There are no significant differences between control and contaminated groups.

Body weight as well as food and drinking water intakes following 9-month chronic ingestion of uranyl nitrate was given in Figure 1. For a better visualization of the different curves, only means were indicated in this figure. The mean ± SD of the control group (noncontaminated) was 24.7 ± 6.0 for drinking water intake (mL/day), 26.9 ± 2.7 for food intake (g/day), and 672 ± 73 for body weight (g), at the end of experiment (9-month contamination). The results show that these chronic contaminations did not affect general health parameters, whatever the uranium content in drinking water (from 0.2 to 120 mg/L) (one-way analysis ANOVA, NS), indicating that these different uranium levels were not toxic for animals following a chronic exposure. In addition, these values allow the calculation of daily intake of uranium throughout the experiment. Dependent on age and body weight, the daily intake of uranium was estimated to be between 10.8 at the beginning of experiment and 3.6 mg/kg body weight per day 9 months later (mean: 5.4 mg/kg/d) for the highest level (120 mg/L). The mean daily intake of uranium was estimated to be 0.009, 0.09, 0.23, 0.45, 0.9, 1.8, and 5.4 mg/kg/d for 0.2, 2, 5, 10, 20, 40, and 120 mg/L, respectively.

Furthermore, no changes in macroscopic appearance or weights of organs (kidney, liver, and spleen) were noted following chronic ingestion by uranium (data not shown).

### 3.2. Uranium Accumulation in Organs

**Figure 2:**
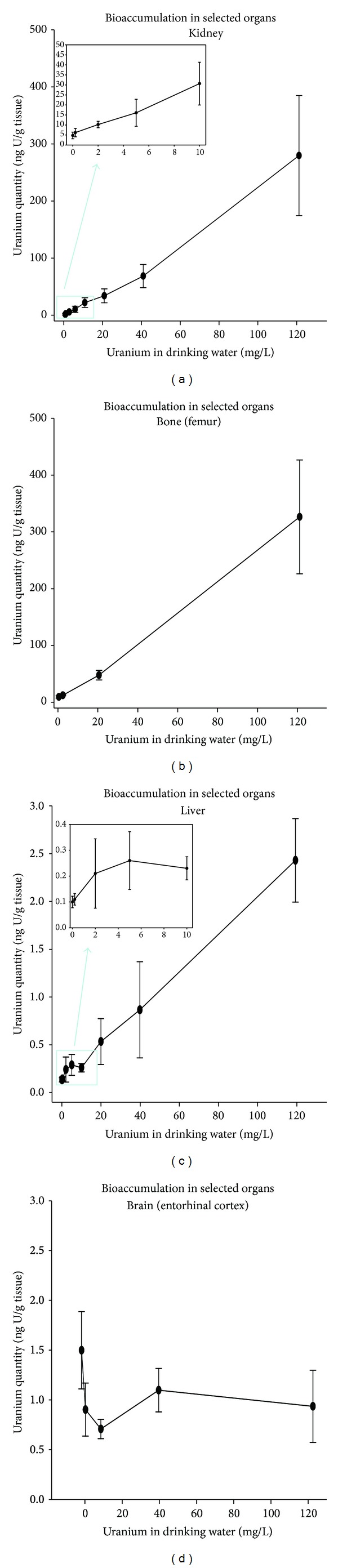
Bioaccumulation of uranium in organs after chronic ingestion for 9 months. Uranium was measured in organs (the kidney, femur, liver, and brain (entorhinal cortex)) by ICP-MS (see Material and Methods section for details). The results are expressed as ng/g tissue for 10 animals in the kidney and femur and for 4–6 in the liver and entorhinal cortex. The insets in the figures on the left (kidney and liver) show the increase in uranium accumulation for the lowest U levels (from 0.2 to 10 mg/L).

Uranium accumulation was measured in different organs after 9-month chronic contamination by ingestion. These measurements were performed in selected organs, that is, the two storage organs of uranium, that is, kidney and bone (femur), as well as liver and brain (entorhinal cortex). Results are indicated in Figure 2. These results show an increasing accumulation of uranium in kidney and bone, depending on the ingested uranium level. These accumulations appeared to be similar in both organs at 120 mg/L. In kidney, the ingestion of the highest level of contamination (120 mg/L) led to a uranium concentration of 352 ng U/g tissue. Concerning the other organs, liver and brain, uranium accumulation was very low as compared to kidney, since the uranium quantity in these tissues was 100-fold less. Uranium quantity in liver is 2.45 ± 0.45 ng/g at 120 mg/L, 0.85 ± 0.51 ng/g at 40 mg/L, and 0.10 ± 0.02 in noncontaminated animals. The uranium accumulation at 120 mg/L corresponds to 2 orders of magnitude less than in the kidneys. The inset linked to the graph representative of uranium accumulation in liver indicates that concentration of 20 mg/L of uranium in drinking water corresponds to a threshold level for uranium accumulation in liver. In central nervous system, the quantities of uranium measured in central nervous system remained extremely low, even at the highest level of 120 mg/L. In fact, there is no dose-dependent accumulation of uranium in brain (0.98 ± 0.34 at 120 mg/L versus 1.5 ± 0.36 ng/g in control group).

### 3.3. Plasma Biochemical Parameters

Plasma biochemical parameters are shown in Table 3. In this table are reported various plasma parameters concerning lipid profile, liver integrity, renal function, and iron metabolism. The results show no changes following a chronic ingestion of uranium for any of these measures (one-way ANOVA analysis, NS). The absence of any difference in plasma concentrations of kidney function markers (creatinine and urea), even at the highest uranium level (120 mg/L), indicates that there was no nephrotoxicity after 9 months of chronic contamination. An increase in the ALAT (3 folds) and ASAT (2 folds) plasma concentrations reflecting liver integrity was noted in the highest group as compared with control group. However, this difference in liver function was not significant, mainly because of great variability in the 120 mg/L group.

### 3.4. Evaluation of Haematopoiesis

**Figure 3:**
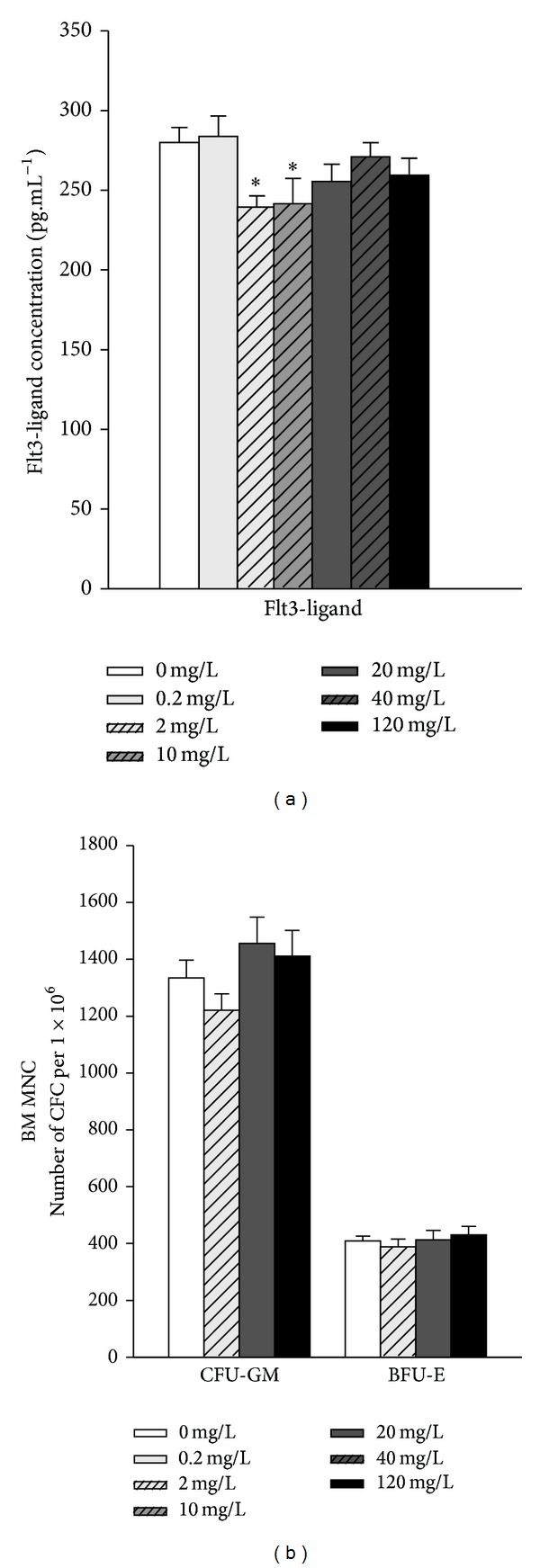
(a) Flt3-ligand in the plasma of animals contaminated through uranium ingestion. A significant difference was observed between 2 and 10 mg/L contaminated animals and control animals (*P* < 0.05, *n* = 10). (b) Progenitor frequency in the bone marrow of control and contaminated animals. Results did not show any significant difference between control and contaminated animals (*n* = 5 animals per group) both for burst forming units erythroid (BFU-E) and colony-forming units-granulocyte macrophages (CFU-GM).

Blood cell counts were performed with differential for granulocytes, lymphocytes, monocytes, platelets, and red blood cells (RBC) (Tables 3 and 4). No significant differences were detected in circulating white blood cells, lymphocytes, granulocytes, or monocytes, regardless of the uranium exposure level. As well, there is no significant difference in RBC numbers, nor in haemoglobin concentration and in haematocrit (Table 3). Two cytokines were also measured in plasma samples, namely, Flt3-ligand and IL-7. The blood level of IL-7 did not vary significantly at any exposure level (data not shown). This indicates that uranium ingestion did not modify T lymphocyte homeostasis at the levels of uranium concentration studied. By contrast, the concentration of the Flt3-ligand was significantly lower in the plasma of animals ingesting 2 and 10 mg/L (Figure 3(a)). This suggested that uranium ingestion induced an increase in haematopoietic activity in these animals, but only in specific contamination conditions, that is, 2 and 10 mg/L of uranium in drinking water. In order to confirm a possible effect of uranium ingestion on haematopoiesis, haematopoietic progenitor frequencies were determined both in the bone marrow and in the spleen of control animals and animals contaminated with 2, 20, and 120 mg/L. Results indicated that uranium ingestion did not induce a modification of progenitor frequency both in the bone marrow (Figure 3(b)) and in the spleen (data not shown). This suggested that the slight modifications in Flt3-ligand concentration observed in the plasma were not associated with a modification of haematopoiesis. Overall, these results suggest that whatever the uranium concentration in drinking water (including the highest uranium concentration of 120 mg/L), uranium ingestion did not modify significantly blood and bone marrow parameters.

### 3.5. Histological Studies

**Figure 4:**
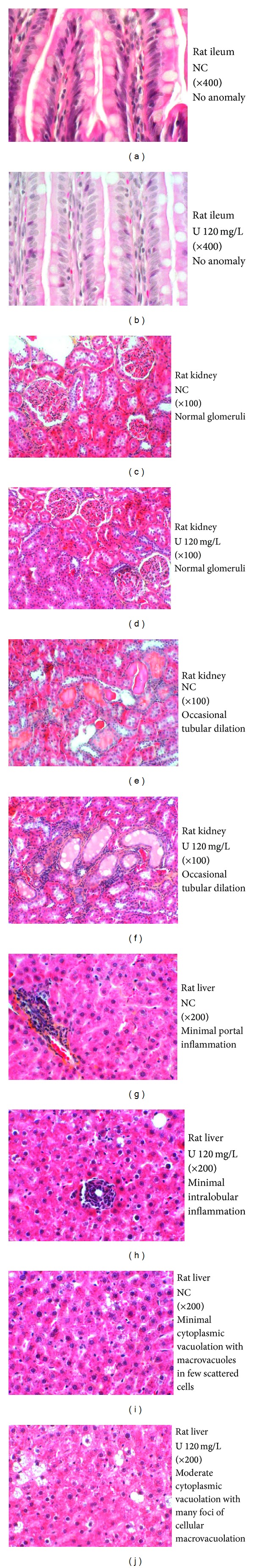
Histological alterations of intestinal, renal, and hepatic sections in rats receiving uranium at 120 mg/L in drinking water for 9 months. Microphotographs of rat tissues were obtained from control (NC: noncontaminated) and contaminated rat (120 mg/L uranium in drinking water). Sections were stained with hematoxylin-eosin-saffron. ((a), (b)) Intestinal longitudinal sections of rat ileum. ((c)–(f)) Histological sections of renal cortex. ((g)–(j)) Liver sections.

Histological analysis of intestine (ileum), liver, and kidney (renal cortex) was performed in control and contaminated animals.  Figure 4 showed microphotographs of histological slides from these organs, and Table 5 reported the different microscopic evaluations for the three organs considered. Concerning the small intestine (terminal ileum), no epithelial injuries were noticed (Figure 4, compare part (a) to (b)). No microscopic anomalies were identified in muscular layers, in nervous structures, or in vessels. Small intestine wall never presented inflammatory alteration or fibrosis. Gut associated lymphoid tissue (GALT) displayed normal microscopic aspect, often with some activation signs (germinal centres). Such lack of histological lesions led to a score equal to zero for intestine regardless the uranium level in drinking water. Concerning kidney microscopic evaluation, glomerular (parts (c) and (d) of the Figure 4) and tubular (parts (e) and (f) of the Figure 4) aspects were analysed. Glomerular lesions were mostly absent (Figure 4, parts (c) and (d)). Only some scattered glomerular cysts could be noticed. Tubulointerstitial lesions remained limited, with minimal to rarely mild intensity when present (Figure 4, parts (e) and (f)). These lesions consisted of multifocal inflammatory cell infiltrates, mainly composed of lymphocytes, with few plasma cells, macrophages, and rare granulocytes. Some tubules were sometimes filled with proteinaceous, eosinophilic material (hyaline casts). Interstitial fibrosis was exceptionally noticed and remained minimal, with only narrow bands of fibrous tissue thickening rare tubular basement membranes and glomerular Bowman's capsules. Tubular necrosis was never observed, whatever the group including the highest level of uranium concentration (120 mg/L). In fact, all these features were recognized in rats of either control or contaminated groups. Tubular regeneration was slightly increased in 10 and 40 mg/L groups, in comparison to control group. Tubular inflammation was slightly increased in 0.2, 10, and 40 mg/L groups, in comparison to control group (Table 5). Surprisingly, this increase remained very limited and was not observed in 120 mg/L group (high dose).

Histological evaluation was also performed in liver (parts (g), (h), (i), and (j) of the Figure 4 and Table 5). Almost all the rats presented inflammatory cell infiltrates into hepatic parenchyma, which always remained minimal and were mainly composed of lymphocytes (mononuclear cells), with few plasma cells, some macrophages, and rare granulocytes. They could be localized in portal areas connective tissue (portal inflammation, Figure 4, part (g)) or appear as scattered and small cellular aggregates slightly distending sinusoids (intralobular inflammation, Figure 4, part (h)). These features were recognized in rats of either control or contaminated groups. Portal necrosis was never observed, whatever the group. Cytoplasmic macrovacuolation was quite regularly observed, with minimal to moderate intensity when present (Figure 4, parts (i) and (j)). Altered hepatocytes displayed in their cytoplasm small vacuoles about 3 to 5 *μ*m in diameter, rounded, well-delimited, with empty lumen. Vacuoles were usually found in small numbers in hepatocytes. Cytoplasmic macrovacuolation was increased in contaminated groups in comparison to control group, particularly in 40 and 120 mg/L groups (Figure 4, part (j)), which suggests that it was related to uranium contamination (Table 5). One-way ANOVA analysis indicates a statistical significant difference between groups (*P* < 0.05).

### 3.6. Intestinal Immune System

**Figure 5:**
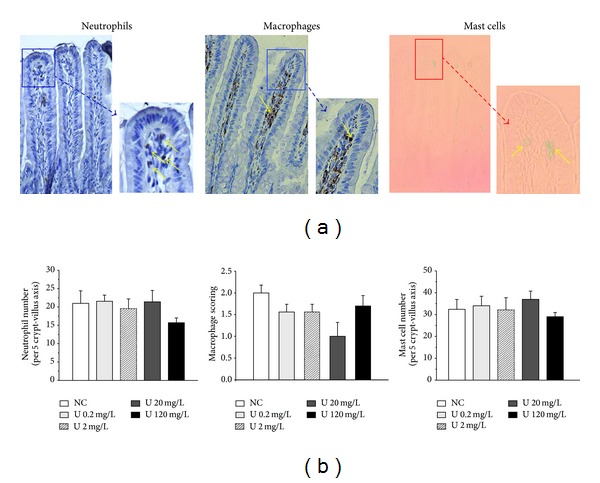
Effects of chronic uranium ingestion on immune cells in the intestinal mucosa in rats receiving contaminated drinking water during 9 months. Micrographs were obtained from control animals (objective ×20 and ×40). The brown staining indicates the cells positive for CD68 (macrophages) and for MPO (myeloperoxidase, neutrophils). Mast cells were stained with Alcian Blue technique. The positive cells were estimated per 5 villus-crypt axes, along the 60 measurements per animal, in control animals (NC: noncontaminated) and in animals contaminated with uranium (U) in their drinking water at various concentrations (0.2, 2, 20, or 120 mg/L). Data were mean ± SD of 8 rats. There is no significant difference between control and experimental groups.

Immune cell composition of lamina propria was investigated after a 9-month chronic ingestion of uranium. This study was performed on neutrophils, macrophages, and mast cells, all of them involved in innate immunity.  Figure 5 shows the effects of uranium on the three selected immune cells after 0.2, 2, 20, and 120 mg/L, as compared with control group (NC: noncontaminated). These results indicated that number of immune cell populations did not vary after 9 months of uranium exposure, at any exposure level (from 0.2 to 120 mg/L). The neutrophil and mast cells number in lamina propria remained constant, even at the highest level (120 mg/L). Concerning macrophages, a decrease in its network in lamina propria seemed to be induced by uranium exposure, with a diminution of 50% at 20 mg/L. However, this decrease was not significant (*P* = 0.069).

**Figure 6:**
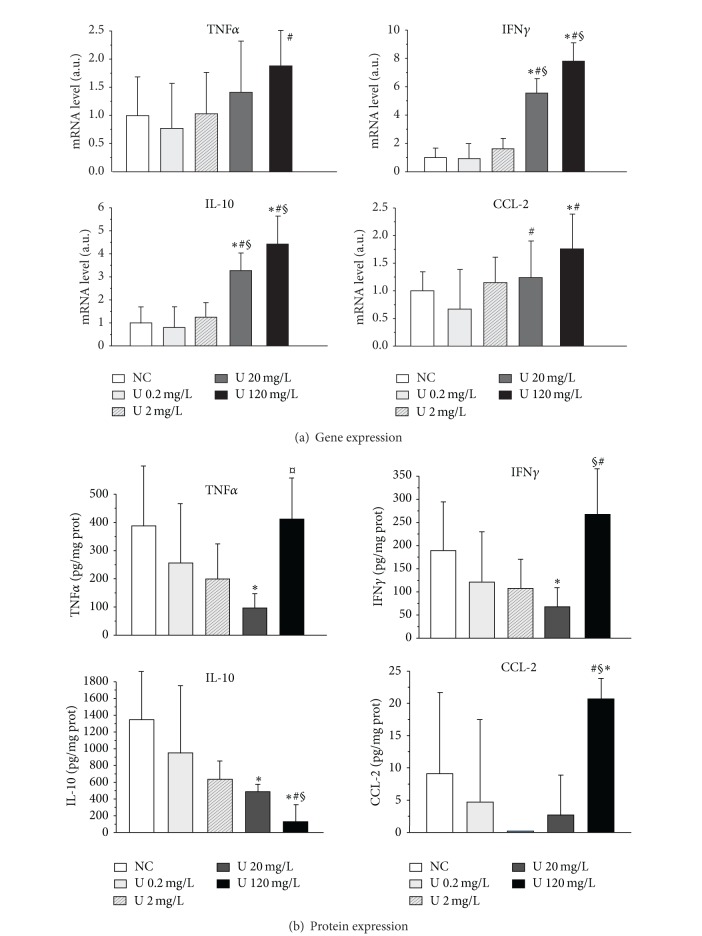
Effects of uranium on cytokine gene and protein expression in rat intestine. Expression was measured in samples from control animals (NC: noncontaminated) and animals contaminated by uranium (U) in their drinking water at various concentrations (0.2, 2, 20, or 120 mg/L). Data are mean ± SD of 9-10 animals. (a) Gene expression: the mRNA levels are expressed as a ratio to the reference gene HPRT (hypoxanthine-guanine phophoribosyltransferase, a housekeeping gene). (b) Protein expression: tissue protein levels are expressed in pg/mg protein. *P < 0.05: significantly different from control values.  ^§^ *P* < 0.05: significantly different from the U 0.2 mg/L group. ^#^ *P* < 0.05: significantly different from the U 20 mg/L group.

To complete this cellular study, effects of uranium were investigated on cytokine and chemokine content in small intestinal wall. The relative mRNA level of 5 cytokines (CCL-2, TNF*α*, IFN*γ*, TGF*β*, and IL-10) was studied in intestinal mucosal extracts of control rats and in rats contaminated at 0.2, 2, 20, and 120 mg/L. The tissue protein level was also studied for CCL-2, TNF*α*, IFN*γ*, and IL-10.  Figure 6 represents the results of gene (Figure 6, top) and protein expression (Figure 6, bottom) for CCL-2, TNF*α*, IFN*γ*, and IL-10. In the top part of Figure 6, we observe an increase in gene expression for the four cytokines studied, which was significant (*P* < 0.05) for uranium levels in drinking water 20 mg/L for IFN*γ*, IL-10, and CCL-2. Modification of TNF*α* gene expression was observed only at 120 mg/L. However, this activation is not general for all cytokines because no changes were observed in the mRNA levels of TGF*β* (data not shown). Experiments were then performed to determine whether these differences in mRNA levels were associated with similar differences in protein levels. The results are indicated in the part (b) of Figure 6. Contrary to cytokine mRNA expression, which increased with uranium exposure, uranium seemed to induce an inhibition of protein levels for three cytokines studied (IFN*γ*, IL-10, and TNF*α*). These variations were also observed for uranium levels >20 mg/L. However, this inhibitory effect was not observed for the highest uranium level, for which a return to control values and even an overincrease were noted for three cytokines (IFN*γ*, CCL-2, and TNF*α*).

### 3.7. Xenobiotic Metabolism in Liver and Kidney

**Figure 7:**
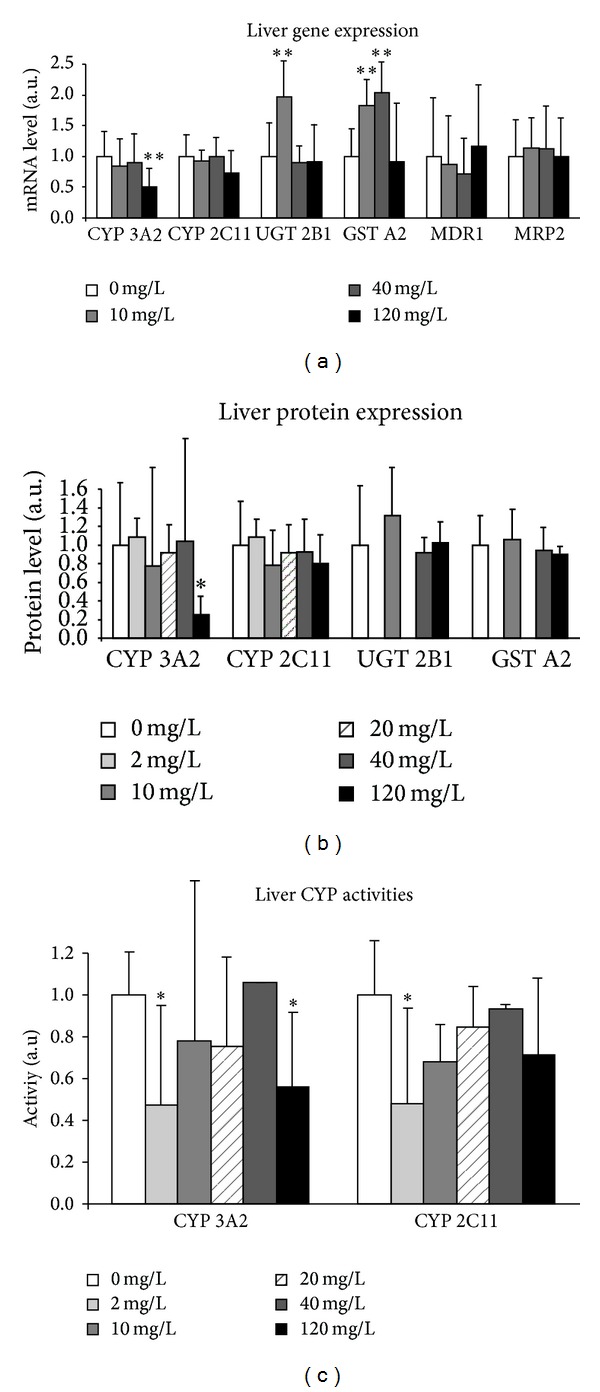
Effects of uranium on enzymes of xenobiotic metabolism in the liver. The molecules of phases I, II, and III of xenobiotic metabolism were measured in control animals (NC: noncontaminated) and in animals contaminated by uranium (U) in their drinking water at various concentrations (10, 40, or 120 mg/L). Gene expression: the mRNA levels are expressed as a ratio to the reference gene HPRT (hypoxanthine-guanine phophoribosyltransferase, a housekeeping gene). Data are mean ± SEM of 8 animals. Protein expression: protein levels are expressed as a ratio to the reference gene GAPDH (glyceraldehyde 3-phosphate dehydrogenase, a housekeeping gene). Data are mean ± SD of 8 animals. CYP activities: enzyme activities are expressed in picomoles per minute per whole liver and values are normalized to the noncontaminated group. Data are mean ± SD of 6 animals. *P < 0.05: significantly different from control values. **P < 0.01: significantly different from control values.

Xenobiotic metabolizing enzymes (XME) were studied in the liver and kidneys, the major organs involved in detoxification. The levels of major enzymes and proteins involved in the three phases of xenobiotic metabolism, phase I (CYP 3A2, CYP2C11), phase II (GSTA2, UGT2B1), and phase III (MRP2, MDR1), were studied in control and uranium-exposed rats.  Figure 7 illustrates the effects of uranium exposure on cytochrome P450, expressed mainly in the liver. Some variations of enzyme activity were observed starting from 2 mg/L of uranium in drinking water, that is, for CYP3A2 (−53%, *P* < 0.05) and CYP2C11 (−52%, *P* < 0.05) (Figure 7(c)), but these effects were not observed at the mRNA and protein levels. By contrast, gene expression of phase II enzymes was modified at 10 (GSTA2, UGT2B1) and 40 mg/L (GSTA2) (data not shown). It appears that 120 mg/L induced major effects, since a drastic decrease in CYP3A2 was observed concomitantly in the levels of mRNA (−50%, *P* < 0.01), protein (−75%, *P* < 0.05), and enzyme activity (−44%, *P* < 0.05) in the rat liver. This indicated that uranium exposure at this concentration altered a major enzyme of the xenobiotic system which could impact the detoxification function in the liver.

**Figure 8:**
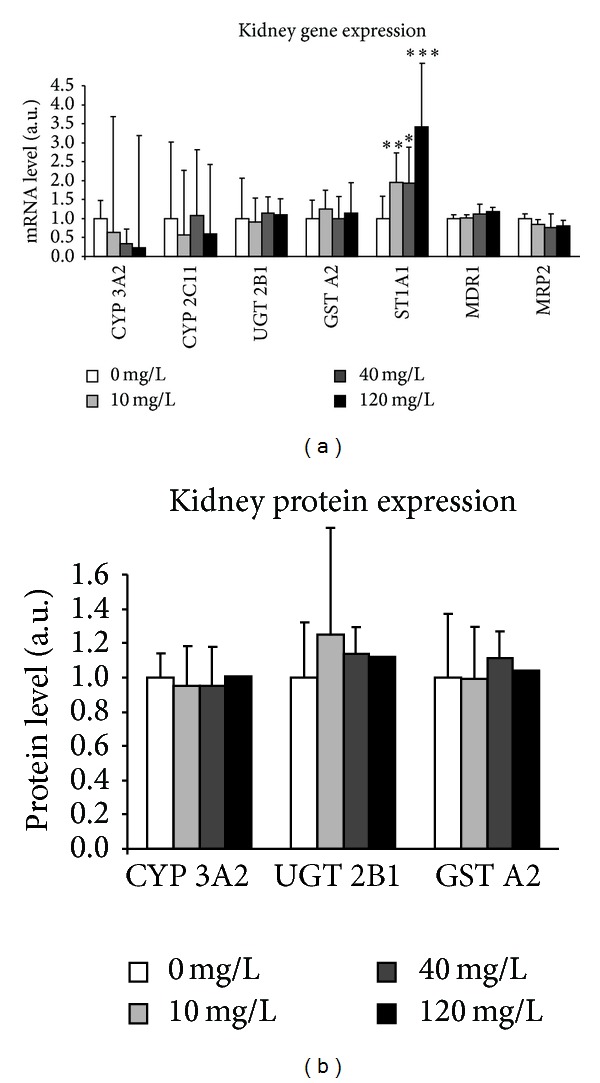
Effects of uranium on enzymes of xenobiotic metabolism in the kidneys. The molecules of phases I, II, and III of xenobiotic metabolism were measured in control animals (NC: noncontaminated) and in animals contaminated by uranium in their drinking water at various concentrations (10, 40, or 120 mg/L). Gene expression: the mRNA levels are expressed as a ratio to the reference gene HPRT (hypoxanthine-guanine phophoribosyltransferase, a housekeeping gene). Data are mean ± SD of 8 animals. Protein expression: protein levels are expressed as a ratio to the reference gene GAPDH (glyceraldehyde 3-phosphate dehydrogenase, a housekeeping gene). Data are mean ± SD of 6 animals. *P < 0.05, **P < 0.01: significantly different from control values. ***P < 0.001: significantly different from control values.

To determine if uranium also targets this detoxification system in the kidneys, the classical storage and target organ of this radionuclide, XME (CYP 3A2 and CYP2C11 for phase I, UGT2B1, GSTA2, and ST1A1 for phase II, and MDR1 and MRP2 for phase III), was studied in the renal cortex.  Figure 8 shows that there was no effect on several phase I (CYP 3A2, CYP2C11) and II (UGT2B1, GSTA2) enzymes. Thus, in contrast to the results observed in liver, this figure indicates that renal mRNA (Figure 8(a)) and protein (Figure 8(b)) levels of CYP3A2 did not change in the kidneys of rats exposed to uranium, at any exposure level. Moreover, there were no changes in UGT2B1 and GSTA2 phase II enzymes, which are more abundant in the kidneys than phase I enzymes. Only an increase, apparently dose-dependent, in STA1 gene expression was observed from 10 mg/L.

### 3.8. Cholesterol Metabolism in Liver

**Figure 9:**
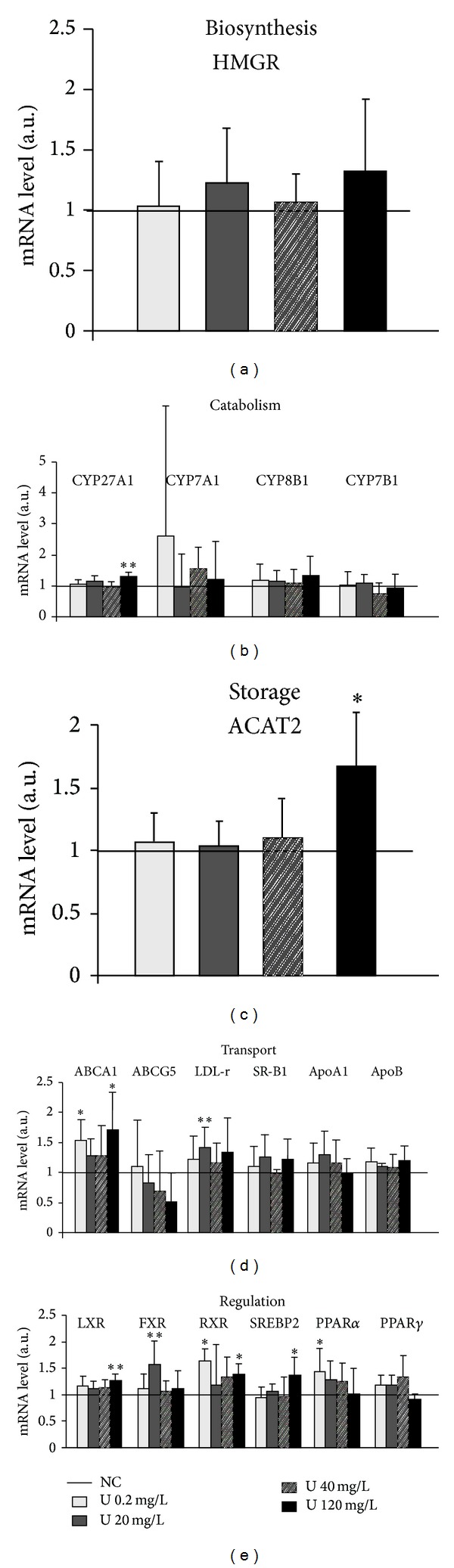
Effects of uranium on gene expression of enzymes of cholesterol metabolism in the liver. The gene expressions were measured in control animals (NC: noncontaminated) and in animals contaminated by uranium in their drinking water at various concentrations (0.2, 20, 40, and 120 mg/L). The mRNA levels are expressed as a ratio to the reference gene HPRT (hypoxanthine-guanine phophoribosyltransferase, a housekeeping gene). Data are mean ± SD of 8 animals. *P < 0.05, **P < 0.01: significantly different from control values.

To complete the evaluation of liver function, we investigated the different steps of cholesterol metabolism in the liver (biosynthesis, catabolism, storage, transport, and regulation). The gene expressions of enzymes and transcription factors involved in this metabolism in the liver of control and contaminated animals with uranium at 0.2, 20, 40, and 120 mg/L were indicated in Figure 9. Any modification was noted for the biosynthesis step. Concerning the catabolism step of cholesterol metabolism, the results show that uranium affected the relative mRNA level of CYP27A1 in the 120 mg/L group (+31%, Figure 9). This highest level of uranium induces also an increase in gene expression of some molecules involved in storage (ACAT2, +70%), in storage (ABCA1, +70%), and in regulation (LXR, RXR, and SREBP2, between 30 and 40%) processes.

**Figure 10:**
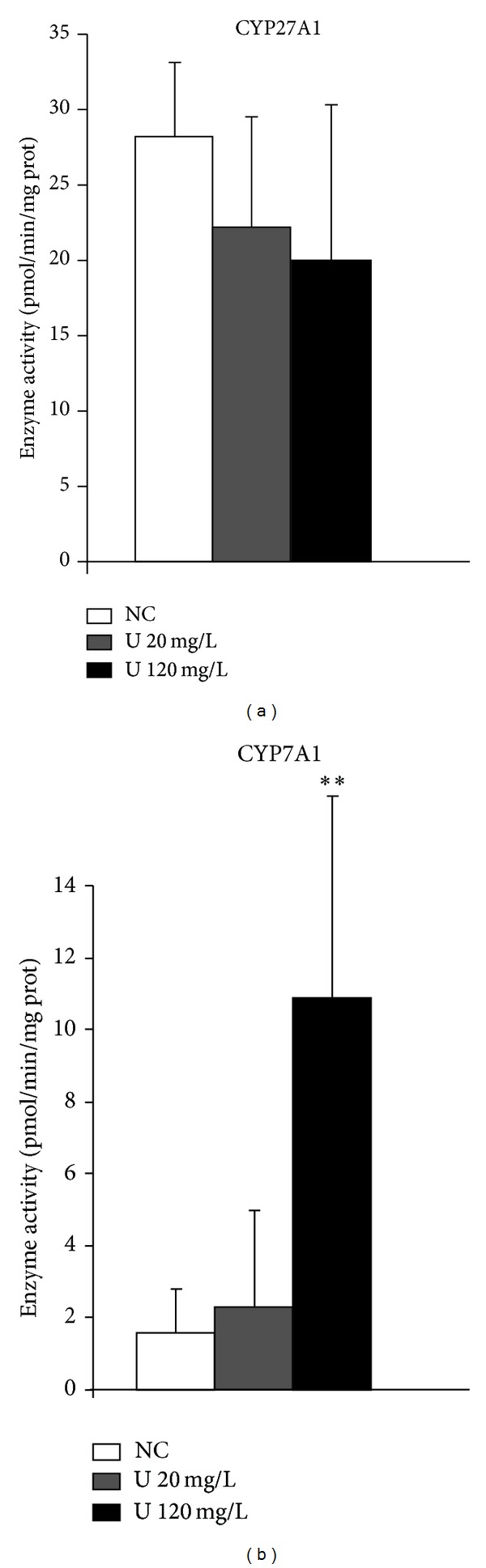
Effects of uranium on enzyme activity of enzymes of cholesterol metabolism in the liver. Enzyme activity is expressed as picomoles per minute per milligram total protein. Data are mean ± SEM of 6 animals. **P < 0.01: significantly different from control values.

These results were completed by measurement of enzyme activity of the two major cytochrome P450 of type 27A1 (CYP27A1) and 7A1 (CYP7A1) (Figure 10). No change was observed for CYP27A1, but uranium affected the activity of CYP7A1 (by a multiple of 6.5, Figure 10).

### 3.9. Cholinergic Pathway in the Entorhinal Cortex

**Figure 11:**
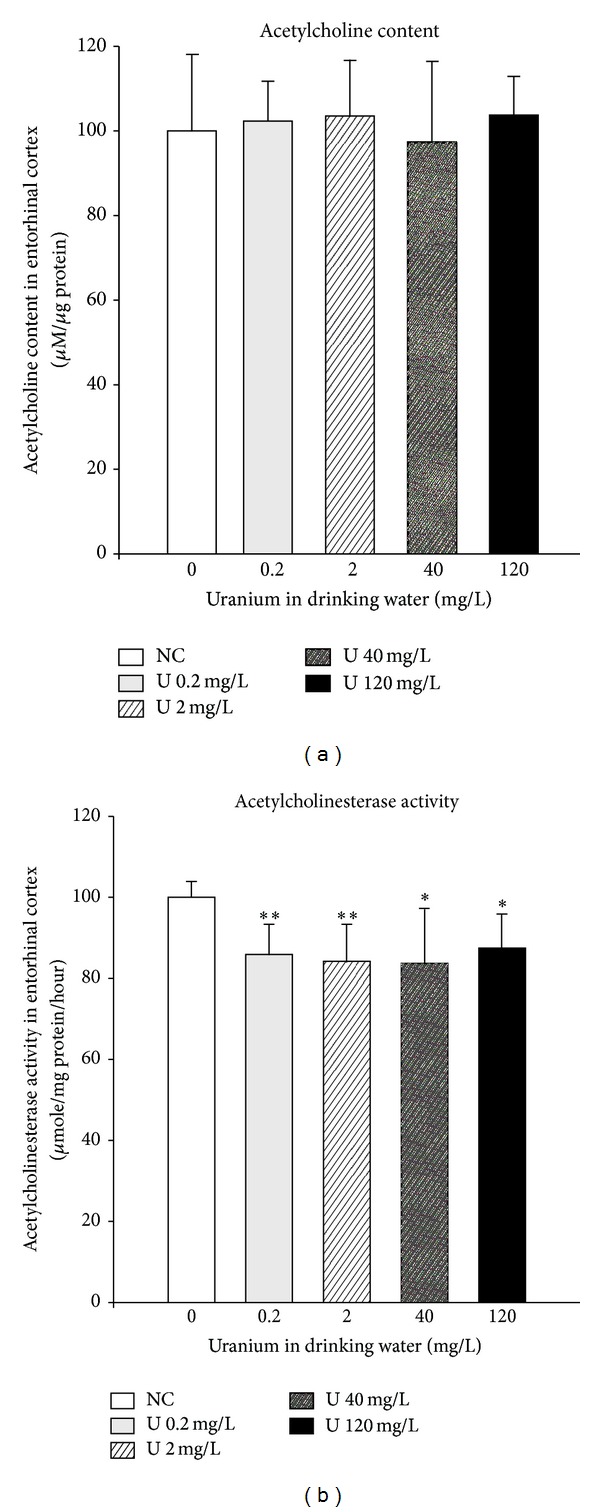
Effect of chronic ingestion of uranium on cholinergic pathway in the entorhinal cortex. The measurements were measured in control animals (NC: noncontaminated) and in animals contaminated by uranium in their drinking water at various concentrations (0.2, 2, 20, 40, and 120 mg/L). Results are expressed as mean ± SD (*n*  =  8–10). *P < 0.05, **P < 0.01: significantly different from control values.

The cholinergic system in the brain (entorhinal cortex) was examined to investigate the molecular effects of uranium that might underlie central nervous system dysfunction.  Figure 11 illustrates the results obtained after a 9-month contamination. Chronic ingestion of uranium did not modify acetylcholine content in the cortex at any exposure level. We did, however, note a dose-independent diminution of acetylcholinesterase activity after uranium contamination (−15%).

### 3.10. Antioxidant Enzymes in the Entorhinal Cortex

**Figure 12:**
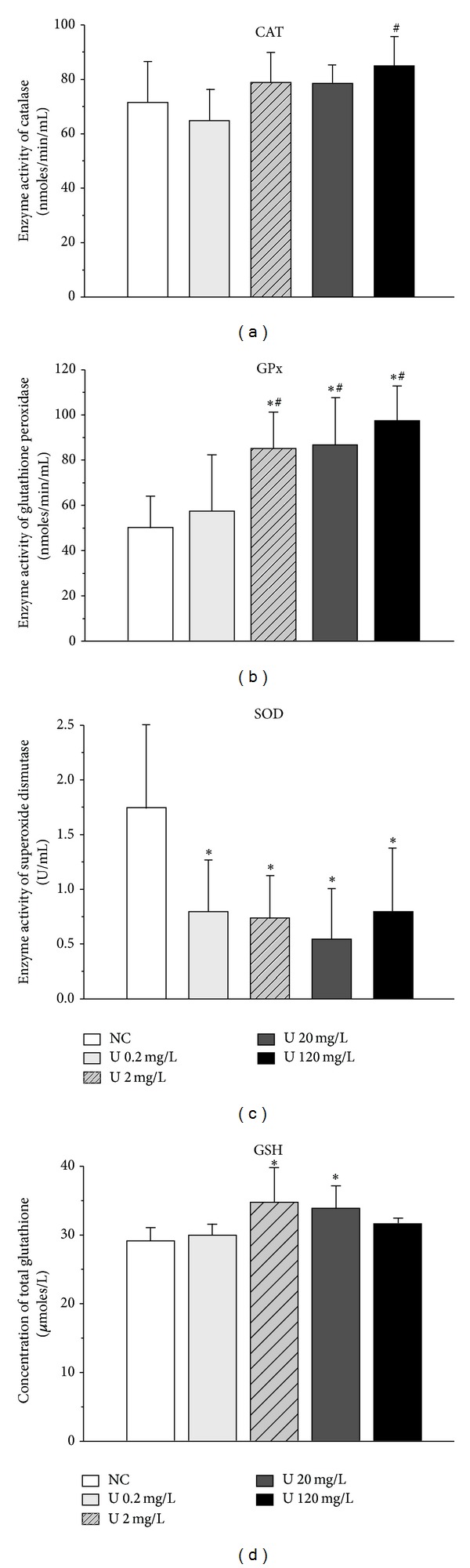
Effect of chronic ingestion of uranium on antioxidant enzyme activity in the entorhinal cortex. Three enzymes were measured, catalase (CAT), glutathione peroxidase (GPx), and superoxide dismutase (SOD), as well as total glutathione (GSH). NC: noncontaminated animal. Results are expressed as mean ± SEM (*n* = 8). *P < 0.05, **P < 0.01: significantly different from control values. ^#^ *P* < 0.05: significantly different from the U 0.2 mg/L group.

Because oxidative stress is a potential mechanism of uranium neurotoxicity, we investigated the effects of uranium on the brain by studying antioxidant defenses. Uranium affected each enzyme studied, but at different thresholds and in opposite directions (Figure 12). It induced a slight increase in catalase activity (+20% at 120 mg/L) and GSH content (+20% at 2 mg/L) and a dose-dependent activation effect on glutathione peroxidase (+68% at 2 mg/L and +90% at 120 mg/L). On the other hand, it inhibited superoxide dismutase activity at all uranium concentrations, in a dose-independent manner (~−50%).

## 4. Discussion

The primary objective of this experimental study was to obtain new data to improve our knowledge of the long-term effects of uranium chronically ingested in drinking water. More specifically, the purpose of this work was to (i) determine the possible thresholds for functional (molecular or cellular) and for pathological (tissue and general health) effects of uranium and (ii) establish the differential sensitivity of the different organs to uranium. The strength of the present study lies in its combination of a wide range of uranium levels (0.2, 2, 5, 10, 20, 40, or 120 mg/L), a large panel of target organs (the small intestine, liver, kidneys, hematopoietic tissue, and brain), and complementary levels of biological analyses (molecular, functional, structural, and pathological).

Concerning the intestines, a uranium accumulation was previously demonstrated in the lamina propria of intestinal wall, near the immune cells [26] and in Peyer's patches [27], a site specialized in the immune response of the small intestine against pathogens. The objective of the present study was thus to investigate uranium effects on immune function of intestinal mucosa due to this specific localization of uranium. The kidneys and the liver are the major organs involved in detoxification processes of endo- and xenomolecules, which constitute in return the primary function of these organs. Thus, the present investigation of uranium effects on the kidneys and the liver was based on the different phases of the xenobiotic metabolism. In addition, cholesterol metabolism was studied in the liver, since this organ plays the central role in whole organism concerning this metabolism. The effects of uranium on the central nervous system were evaluated *via* two mechanisms, the uranium-induced oxidative stress and the cholinergic system. Indeed, a possible mechanism of uranium effects observed in cognitive functions may be due to the oxidative stress induced by reactive oxygen species imbalance [30]. A uranium-induced chronic cerebral oxidative stress may have subsequent consequences on brain function, with possible development of neurological disorders [39]. Effects of uranium on cholinergic system may lead to impairment in learning and attention [40] or promote neurodegenerative pathologies such as Alzheimer's disease. Finally, effects induced by uranium were measured in hematopoietic cells, since uranium accumulated in the bones and kidneys following chronic contamination [25, 41].

As indicated above, this study investigated several parameters of specific function(s) of all organs considered, to ensure the absence of possible consequences of uranium exposure on them. The present study did demonstrate that uranium induces some effects at the molecular level on the five considered organs.

Investigation of cholesterol metabolism in the liver indicated variations of mRNA levels for the molecules involved, but most uranium-induced effects were noted at 120 mg/L and led to minor modifications in catabolism, storage, transport, and regulation processes. These results indicate that uranium targets cholesterol metabolism at mRNA level, as previously reported [42]. Nevertheless, these biological effects were not harmful as they had no impact on the plasma lipid profile. Concerning the liver xenobiotic metabolism, some slight effects on mRNA levels were reported at low exposure (2 mg/L) and more important effects (mRNA, proteins, and activity) on a specific enzyme (CYP3A) with a greater variation at high exposure (120 mg/L). These results supplemented previous studies of xenobiotic metabolism in the liver [23, 36]. Surprisingly, the present study did not observe these effects on xenobiotic metabolism in the kidney, the organ considered most sensitive to uranium. This lack of molecular effects on the kidneys is in accordance with previous results obtained by Linares et al., which demonstrated induction of oxidative stress only at uranium levels above 400 mg/L [43]. In the small intestine, changes of protein and mRNA tissue levels of cytokines were observed for medium (20 mg/L) and high (120 mg/L) uranium levels, demonstrating effect of uranium on inflammatory mediators in the intestines, corroborating the hypothesis that the intestinal system may be a biological target of uranium following ingestion. However, these variations should not be considered adverse effects, given that the number of immune cells in the intestinal mucosa did not change significantly. The only change was a nonsignificant 50% diminution of macrophages, as mentioned previously [20]. Biological effects of uranium on the brain were estimated by investigating the cholinergic pathway and oxidative stress. For the former, a non-dose-dependent reduction in acetylcholinesterase activity began at 0.2 mg/L, the lowest exposure level. Such decreased acetylcholinesterase activity was already reported [38]. Modifications of the activity of antioxidant enzymes were also observed at that level. At higher exposures (2, 20, and 120 mg/L), the activity of 3 enzymes (of the 4 studied) was modified. Other recent studies have reached the same conclusions, reporting opposite effects by uranium on the antioxidant status of the brain, depending on radiological activity [30] and uranium levels [44]. As for the other organs, these modifications need not be considered deleterious, since both antioxidant enzyme activity and acetylcholine levels were not reduced in the brain. Finally, no real damages to hematopoiesis were observed. This finding suggests that the slight modifications of the Flt3-ligand concentration in the blood were not linked to a major modification of hematopoietic activity.

Overall, these results indicate that uranium ingestion is able to induce subtle but significant effects at the molecular level, mainly on mRNA expression, which does not have any deleterious consequences.

Different thresholds may be deduced on the basis of these observed molecular effects: 20 mg/L for the intestine, >10 mg/L for the kidneys, >2 mg/L for the liver, and only 0.2 mg/L for the brain. This indicates that the kidneys are not the organ the most sensitive to chronic contamination by uranium ingestion. The brain appears very sensitive, as several previous studies have shown [44–46]. At high doses, uranium may induce adverse effects including impairment of memory [47] and locomotion [45]. Surprisingly, these results demonstrated that there is no clear positive relationship between uranium accumulation and its biological effects. Indeed, this relation appeared to be an inverse one: the organs the most affected by uranium exposure were those that did not accumulate it (1 ng/g in brain, 2.5 ng/g in liver, and 350 ng/g in kidney, at 120 mg/L). These values are consistent with data of previous studies that indicated no real accumulation of uranium in central nervous system following chronic contamination with uranium [30, 48].

Such observation was confirmed in other organs, namely, gonads. Indeed, very low quantities of uranium were measured in ovaries [49, 50] and testis [43], when uranium effects were observed on reproductive function. This finding shows the special sensitivity of the brain or the gonads to chemical toxicity by metals, notably uranium [51].

Another noteworthy point about the observations in this study is the shape of the dose-response relations. Although we might have expected increased classical dose-response curves, we found also that uranium contamination elicited U-shaped or inverted U-shaped curves. Such nonmonotonic shapes have previously been described following chronic low-dose uranium contamination. A kinetic study performed during a chronic ingestion of uranium in drinking water showed successive waves of uranium accumulation and elimination depending on the time exposure [48]. Nonmonotonic curves were also observed with uterine parameters depending on uranium levels [52]. Two nonexclusive hypotheses may be proposed. Firstly, it can be assumed that uranium may inhibit or activate the concerned genes depending on the dose. Secondly, the number and the role of molecular targets for uranium may vary with exposure levels, modifying thus the whole response of the organ. Only the elucidation of the underlying mechanisms of uranium effects following a chronic exposure to low level could reply to these queries. Besides, the nonmonotonic form of these dose-response relations raises the problem of their use for the possible establishment of guidelines [53].

The second major information of the present study concerns the nondeleterious effects induced by uranium. This present study demonstrates clearly that chronic ingestion of uranium at environmental exposures (≤2 mg/L) and higher (up to 120 mg/L) did not produce harmful effects in rats, as evidenced by the absence of clinical signs and histological lesions in all the organs we studied. Our study observed only a trend toward minor uranium-induced histological impairment, specifically, increased cytoplasmic vacuolation in the liver. Gilman and his colleagues reported also this minor histological alteration in the kidneys and in the liver at all uranium exposure levels [19]. The lack of adverse histological lesions in the kidneys is in accordance with the absence of changes of urea and creatinine in blood, traditionally considered as bioindicators of a renal dysfunction. The primary site of accumulation and subsequent effects of uranium in the kidneys was the proximal tubule cells as observed with other metals [54]. Specific biomarkers of proximal tubule function were not analyzed in this study, but a recent study [36] failed to evidence a change of Kim-1, kallikrein, and osteopontin levels following a chronic exposure to uranium with a contamination protocol (9 months with 40 mg/L) similar to this used in the present study. This point strengthens the absence of marked uranium effects in the kidneys even at high level. In fact, the maximal uranium quantity measured in kidney of the present study following a chronic exposure at the highest level corresponded to 1/10 of the nephrotoxic threshold of 3 *μ*g/g defined for an acute exposure [15], despite a high uranium level in drinking water. This may explained the absence of adverse effects in kidneys. Our conclusion is consistent with other reports, including the recent study by Linares et al. [43], which demonstrated only minor histological lesions for uranium exposure >200 mg/L. However, some studies are not in accordance with the present study and the studies cited above. For instance, these results are not concordant with those obtained by Gilman et al. in 1998 [19]. The objective of Gilman's study was to determine a NOAEL in rats by investigating kidney and liver after chronic ingestion of uranium-contaminated drinking water (from 0.96 to 600 mg/L). The authors reported adverse renal lesions on tubules and glomeruli. Surprisingly, these histological lesions were observed in all contaminated groups including the lowest exposure of 0.96 mg/L, independently of dose. It is noteworthy that no other experimental study has been performed at such low exposure levels. Other publications have noted histological lesions of rat kidneys, but at higher uranium exposure levels (200 mg/L, [43]; 30 mg/L, [55]). Recently, Homma-Takeda et al. published deleterious effects of uranium in different rat models (neonate, prepubertal, and adult) after subcutaneous administration of uranium acetate [56]. The discrepancy is very likely due to the difference of the administration mode. Indeed, previous study demonstrated that only 0.5% of uranium present in drinking water was absorbed into blood by the gastrointestinal system [57].

This lack of histopathological lesions and clinical signs was thus in accordance with the subtle biological effects observed in animals contaminated with uranium regardless the uranium level in drinking water.  Table 6 summarizes the data obtained in the present study, biological effects and deleterious effects.

Thus, according to standard criteria, we conclude that the NOAEL (No-Observed-Adverse-Effect Level) threshold for uranium chronically ingested in drinking water is superior to 120 mg/L for male adult rats. The findings of the present study indicate thus that the NOAEL threshold based in the present study on the histological alterations was >125 times higher than the level determined in Gilman's study (>120 mg/L versus <0.96 mg/L, resp.).

The guideline issued by WHO (2005) [3] derived with a TDI (tolerable daily intake) approach from Gilman's study (1998) [19] and based on uranium's chemical toxicity to the kidneys assessed a LOAEL of 0.96 mg/L, equivalent to 0.06 mg/kg/day. This TDI, combined with a presumed uncertainty factor of 100 and a presumed 60 kg adult consuming 2 L of drinking water daily, yielded a guideline value of 15 *μ*g/L (before July 2011). A similar calculation was applied to our results deduced from a 5.4 mg/kg/day dose ingested by a rat weighting between 300 and 700 g during the whole experiment and consuming between 20 and 30 mL of water contaminated at 120 mg/L (see Figure 1). This calculation leads to a guideline value of 1350 *μ*g/L, a very high value compared with environmental levels and the WHO reference guideline. Indeed, WHO established a new guideline value in 2011 based on new epidemiological studies not able to demonstrate obvious effects of uranium below an exposure concentration of 30 *μ*g/L [17]. In fact, changes of proximal tubular function of kidneys remained in the physiological range for the different published epidemiological studies for uranium levels <30 *μ*g/L [18]. Our results seem consistent with epidemiological studies that found no adverse effects in adults exposed to drinking-water containing naturally high uranium levels of 500 to 1000 *μ*g/L [58–61]. Other studies have reported some slight effects on renal function for uranium exposure >300 *μ*g/L [62, 63], effects that did not appear either deleterious or irreversible. The conjunction of experimental and epidemiological studies suggests thus that the threshold for induction of renal adverse effects should at least be above 300 *μ*g/L for humans consuming uranium-contaminated drinking water. This value was 10-fold higher that the guideline value of 30 *μ*g/L provided by WHO in 2011 [17].

## 5. Conclusions

In conclusion, TDI calculation leads to a guideline value of 1350 *μ*g/L, a very high value compared with WHO reference guideline (30 *μ*g/L). Our experimental results associated with published epidemiological studies suggest that adverse chemical effects of uranium on kidneys in humans should be expected only for values above 300 *μ*g/L. In light of these new results, it appears that the current WHO reference guideline for uranium content in drinking water is very protective (×100) and might be reconsidered.

## Figures and Tables

**Table 1:** Determination of uranium radioisotopes present in drinking water contaminated with 40 mg/L.

Uranium		
^234^U	Bq/L	93.4 ± 6.5
^235^U	Bq/L	8.70 ± 1.13
^238^U	Bq/L	531.5 ± 31.9

U total	mg/L	42.85 ± 2.57
Ratio ^235^U/^238^U	%	0.25

Uranium was analysed by alpha spectrometry following chemical separation. See Material and Methods section for more details.

**Table 2:** Primer sequences for rat genes studied in several organs following chronic ingestion of uranium.

Genes	Forward	Reverse
HPRT	5′-GCTCGAGATGTCATGAAGGAGA-3′	5′-TCAGCGCTTTAATGTAATCCAGC-3′

	Cytokines	
CCL-2	5′-CAGCCAGATGCAGTTAATGCC-3′	5′-AGCCGACTCATTGGGATCAT-3′
TNF*α*	5′-ATCCGAGATGTGGAACTGGC-3′	5′-CGATCACCCCGAAGTTCAGTA-3′
IFN*γ*	5′-CACGCCGCGTCTTGGT-3′	5′-TCTAGGCTTTCAATGAGTGTGCC-3′
TGF*β*	5′-TCCCAAACGTCGAGGTGAC-3′	5′-CAGGTGTTGAGCCCTTTCCA-3′
IL-10	5′-GTTGCCAAGCCTTGTCAGAAA-3′	5′-TTTCTGGCCATGGTTCTCT-3′

	Xenobiotic metabolising enzymes	
CYP3A2	5′-AGTAGTGACGATTCCAACATAT-3′	5′-TCAGAGGTATCTGTGTTTCCT-3′
CYP2C11	5′-ATGGGATGCAATGGAAGGAG-3′	5′-TCTTGCCCATCCCAAAAGTC-3′
ST1A1	5′-AGGGTGGCAAGCTAGAGAAGTG-3′	5′-GAGGGAACCCCTGGACATTT-3′
GSTA2	5′-TTGACATGTATTCAGAGGGT-3′	5′-TTGTTTTGCATCCATGGCTG-3′
UGT1A1	5′-TGGCATCCCCAAAACGATCT-3′	5′-GGAACCGGAGTGTGTGATGAA-3′
UGT2B1	5′-TGGAGAACATGGTGTAGTGGT-3′	5′-TTGGCTTTTTCTTCAGTAGTCAGG-3′
MDR1	5′-ATCAACTCGCAAAAGCATCC-3′	5′-AATTCAACTTCAGGATCCGC-3′
MRP2	5′-TTCGAAGCTGGATGATGTGTTT-3′	5′-GCCATGCAGATCATGACAAGAG-3′

	Enzymes of cholesterol metabolism	
HMGR	5′-GGCGGGTCCTGCAAGTG-3′	5′-GCAGGTGAGCGGGTGAGA-3′
CYP27A1	5′-GGAAGGTGCCCCAGAACAA-3′	5′-GCGCAGGGTCTCCTTAATCA-3′
CYP7A1	5′-CCAAGTCAAGTGTCCCCCTCTA-3′	5′-GACTCTCAGCCGCCAAGTG-3′
CYP8B1	5′-GTACACATGGACCCCGACATC-3′	5′-GGGTGCCATCAGGGTTGAG-3′
CYP7B1	5′-TCAGATGCAAAGACGGTCAGA-3′	5′-TTCATGCCCGTAGTATTTTTTCAG-3′
ACAT2	5′-GCCCCAGCCGACATTTT-3′	5′-GTGCAGTGTGAAGCCTTGACTT-3′
ABCA1	5′-ATCTCATAGTATGGAAGAATGTGAAGCT-3′	5′-CGTACAACTATTGTATAACCATCTCCAAA-3′
ABCG5	5′-CGCAGGAACCGCATTGTAA-3′	5′-TGTCGAAGTGGTGGAAGAGCT-3′
LDL-r	5′-CAGCCGATGCATTCCTGACT-3′	5′-AGTTCATCCGAGCCATTTTCAC-3′
SR-B1	5′-GTTGGTCACCATGGGCCA-3′	5′-CGTAGCCCCACAGGATCTCA-3′
ApoA1	5′-AATGGGACAGGGTGAAGGA-3′	5′-TGAACCCAGAGTGTCCCAGTT-3′
ApoB	5′-TCCTAACATCATTGTGCCTTCAT-3′	5′-CCTTGAAATCTGGGAGGGAAAACT-3′
LXR*α*	5′-AGCAACAGTGTAACAGGCGCT-3′	5′-GTGCAATGGGCCAAGGC-3′
FXR	5′-TGACAAAGAAGCCGCGAAT-3′	5'-TGTAATGGTACCCAGAGGCCC-3′
RXR*α*	5′-CGCAAAGACCTGACCTACACC-3′	5′-TCCTCCTGCACGGCTTCCC-3′
SREBP2	5′-AGCTGGCAAATCAGAAAAACAAG-3′	5′-CGATCTTCAAGTCCACATCACTGT-3′
PPAR*α*	5′-TCTCTTCCCAAAACTCCTTCA-3′	5′-GCACGAGCTGCGCATGCTC-3′
PPAR*γ*	5′-TCA TGA CCA GGG AGT TCC TCA-3′	5′-TCATCTAATTCCAGTGCATTGAACTT-3′

**Table 3:** Plasma parameters in rats contaminated to uranium in drinking water for 9 months.

Parameters	Noncontaminated	Uranium (mg/L)				
		0.2	10	20	40	120
Glycaemia						
Glucose (mM)	11.58 ± 1.14	11.1 ± 1.17	10.51 ± 1.36	11.74 ± 1.52	9.91 ± 0.95	10.63 ± 1.23
Lipids						
Cholesterol (mM)	1.99 ± 0.54	2.3 ± 0.63	2.33 ± 0.38	2.14 ± 0.89	2.40 ± 0.82	2.43 ± 0.76
LDL-cholesterol (mM)	0.38 ± 0.51	0.46 ± 0.22	0.48 ± 0.13	0.41 ± 0.73	0.50 ± 0.35	0.36 ± 0.38
HDL-cholesterol (mM)	1.40 ± 0.41	1.59 ± 0.38	1.65 ± 0.32	1.54 ± 0.63	1.62 ± 0.51	1.58 ± 0.89
Phospholipids B (g/L)	1.38 ± 0.25	1.54 ± 0.28	1.56 ± 0.16	1.59 ± 0.44	1.64 ± 0.38	1.72 ± 0.44
Triglycerides (mM)	1.27 ± 0.47	1.60 ± 0.73	1.53 ± 0.47	2.0 ± 0.92	1.59 ± 0.57	1.74 ± 0.54
Liver integrity						
ALAT (U/L)	43.4 ± 12.3	43.0 ± 16.1	37.6 ± 11.7	38.0 ± 10.75	37.8 ± 19.6	139.9 ± 183.0
ASAT (U/L)	118.1 ± 38.9	108.6 ± 35.4	93.1 ± 27.5	98.4 ± 26.9	96.1 ± 42.7	231.9 ± 225.0
Bilirubin (µM)	55.6 ± 4.74	60.7 ± 3.5	61.0 ± 7.6	63.2 ± 10.1	58.0 ± 4.4	60.9 ± 4.7
Renal function						
Creatinine (µM)	49.3 ± 3.32	50.2 ± 4.11	50.4 ± 4.74	52.0 ± 4.74	47.0 ± 4.4	48.9 ± 5.1
Urea (mM)	5.27 ± 0.82	5.22 ± 0.82	5.47 ± 0.89	5.81 ± 1.04	4.81 ± 0.82	5.30 ± 0.95
Iron metabolism						
Iron (µM)	31.5 ± 7.91	26.6 ± 3.16	33.4 ± 7.27	33.3 ± 5.7	27.6 ± 5.4	30.3 ± 8.5
Ferritin (ng/L)	31.1 ± 13.0	36.0 ± 21.5	39.3 ± 18.34	21.3 ± 11.4	20.3 ± 13.9	23.9 ± 18.3
Transferrin (g/L)	1.39 ± 0.13	1.49 ± 0.19	1.53 ± 0.16	1.55 ± 0.38	1.58 ± 0.22	1.60 ± 0.19
Ceruloplasmin (mg/L)	72.3 ± 8.5	70.2 ± 28.8	81.6 ± 13.6	63.5 ± 19.3	89.1 ± 17.4	90.4 ± 24.4
Red blood cells (×10^12^/L)	9.04 ± 1.30	9.48 ± 1.9	9.28 ± 1.1	9.28 ± 2.0	9.74 ± 2.0	8.90 ± 4.6
Hematocrit (%)	43.9 ± 8.9	46.8 ± 14.2	46.4 ± 9.8	45.5 ± 8.2	47.8 ± 11.1	42.7 ± 20.6
Hemoglobin (g/L)	14.2 ± 2.21	14.7 ± 1.9	14.6 ± 2.53	14.7 ± 1.9	14.9 ± 1.9	13.8 ± 7.0

Mean ± SD, *n* = 10 per group. There are no significant differences between groups (one-way ANOVA with *P* < 0.05 significant).

**Table 4:** Blood cell counts and differential in rats ingesting uranium through drinking water during 9 months.

Cell lineage (×10^9^ per L of blood)	Noncontaminated	Uranium (mg/L)				
		0.2	10	20	40	120
White blood cells	4.43 ± 1.58	6.19 ± 1.01	5.83 ± 2.72	5.40 ± 1.83	5.31 ± 1.64	6.20 ± 4.11
Lymphocytes	3.22 ± 1.23	3.20 ± 1.39	7.45 ± 1.33	5.85 ± 1.11	4.06 ± 1.33	3.70 ± 1.83
Granulocytes	1.07 ± 0.54	1.67 ± 0.35	1.52 ± 0.70	1.32 ± 0.28	1.15 ± 0.41	2.35 ± 2.43
Monocytes	0.08 ± 0.03	0.13 ± 0.03	0.12 ± 0.06	0.10 ± 0.03	0.10 ± 0.03	0.15 ± 0.13
Platelets	472 ± 484	603 ± 847	229 ± 351	177 ± 395	455 ± 496	165 ± 405

Mean SD, *n* = 9-10 per group. There are no significant differences between groups (one-way ANOVA with *P* < 0.05 significant).

**Table 5:** Histological alterations in intestine, kidney, and liver from rats contaminated with uranium in drinking water for 9 months.

Parameters	Noncontaminated	Uranium (mg/L)			
		0.2	10	40	120
Intestine					
Villous epithelial injury	0	0	0	0	0
Villous atrophy	0	0	0	0	0
Crypt hyperplasia	0	0	0	0	0
Crypt distension	0	0	0	0	0
Goblet cell hyperplasia	0	0	0	0	0
Inflammation	0	0	0	0	0
Mucosal fibrosis	0	0	0	0	0
Kidney					
Mesangial proliferation/glomerulosclerosis	0	0	0	0	0
Glomerular cystic dilation	0.08	0	0.1	0	0
Tubular necrosis	0	0	0	0	0
Tubular regeneration	0.67	0.5	0.8	0.75	0.4
Tubular dilation	0.67	0.6	0.4	0.33	0.3
Tubular inflammation	0.83	1	0.9	1	1
Interstitial fibrosis	0.42	0.1	0.3	0.33	0
Liver					
Portal inflammation	1	1	1	1	1
Intralobular inflammation	0.92	0.9	1	0.67	0.9
Periportal necrosis	0	0	0	0	0
Intralobular necrosis	0	0	0	0	0
Cytoplasmic vacuolation	0.67	1.2	1.4	1.67*	2.2*
Fibrosis	0	0	0	0	0

Intestinal lesions scoring: group means (scores/3), with *n=6*–8.

Renal lesions scoring: group means (scores/4), with *n=5*-6.

Hepatic lesions scoring: group means (scores/4), with *n=5*-6.

*P < 0.05 between groups (one-way ANOVA analysis).

**Table 6:** Symopsis of uranium accumulation and effects in rats contaminated with uranium in drinking water for 9 months.

Uranium (mg/L)	0.2	2	5	10	20	40	120
Health	Type equation here.						
Body weight	=	=	=	=	=	=	=
Water consumption	=	=	=	=	=	=	=
Food consumption	=	=	=	=	=	=	=
Intestine							
Gene expression of cytokines	=	=			↑	=	↑
Protein expression of cytokines	=	=			↓	=	↑
Immune cell number	=	=			=	=	=
Histology	=	=		=	=	=	=
Kidney							
Uranium accumulation		↑	↑	↑	↑	↑	↑
Gene expression of EMX				↑		↑	↑
Protein expression of EMX				=		=	=
Histology	=	=	=	=	=	=	=
Plasma parameters	=	=	=	=	=	=	=
Liver							
Uranium accumulation	=	=	=	=	↑	↑	↑
Gene expression EMX				↑		↑	=
Protein expression EMX		=		=	=	=	↓
Enzyme activity EMX		↓		=	=	=	↓
Gene expression Chol Met	=				=	=	↑
Enzyme activity Chol Met					=		↑
Histology	=	=	=	=	=	↑	↑
Plasma parameters	=			=	=	=	=
Haematopoiesis							
Uranium accumulation in bone		=			↑		↑
Bone marrow progenitors	=				=		=
Spleen progenitors	=				=		=
Blood cell counts	=			=	=	=	=
Plasma cytokines	=	↓	=	↓	=	=	=
Brain							
Uranium accumulation		=		=		=	=
Antioxidant enzymes	↓	↓↑			↓↑		↓↑
Cholinergic pathway	=↓	=↓				=↓	=↓

EMX: enzymes and transcription factors of xenobiotic metabolism; Chol Met: enzymes and transcription factors of cholesterol metabolism.

## Abbreviations

DU: Depleted uranium
EMX: Enzymes of xenobiotic metabolism
NOAEL: No-Observed-Adverse-Effect Level
LOAEL: Low-Observed-Adverse-Effect Level
LOEL: Low-Observed-Effect Level
TDI: Tolerable daily intake
WHO: World Health Organization.
